# BDNF-Related Imbalance of Copine 6 and Synaptic Plasticity Markers Couples With Depression-Like Behavior and Immune Activation in CUMS Rats

**DOI:** 10.3389/fnins.2018.00731

**Published:** 2018-10-31

**Authors:** Yin-xiu Han, Chen Tao, Xin-ran Gao, Le-le Wang, Fu-hao Jiang, Chong Wang, Ke Fang, Xing-xing Chen, Zheng Chen, Jin-fang Ge

**Affiliations:** ^1^School of Pharmacy, Anhui Medical University, Hefei, China; ^2^Anhui Province Key Laboratory of Major Autoimmune Diseases, Anhui Institute of Innovative Drugs, Hefei, China; ^3^The Key Laboratory of Anti-inflammatory and Immune Medicine, Ministry of Education, Anhui Medical University, Hefei, China

**Keywords:** depression, chronic unpredictable mild stress (CUMS), saccharin preference, Copine 6, BDNF, synaptotagmin I, synapsin I

## Abstract

Chronic stress is a contributing risk factor in the pathogenesis of depression. Although the mechanisms are multifaceted, the relationship can be ascribed partly to stress-related alterations in immune activation and brain plasticity. Considering the increasing evidence regarding the role of Copine 6 in the regulation of synaptic plasticity, the aim of the present study is to investigate Copine 6 expression in the hippocampus and the prefrontal cortex (PFC) in a stress-induced depression rat model. The behavior of the rats was evaluated via the open field test, saccharin preference test, elevated plus maze test, tail suspension test, Morris water maze, and forced swimming test. The plasma concentrations of C-reactive protein (CRP) and interleukin-6 (IL-6) were measured, and the protein expressions of brain-derived neurotrophic factor (BDNF), Copine 6, and synaptic plasticity markers in the hippocampus and the PFC were also detected. The results showed that chronic unpredictable mild stress (CUMS) induces depression-like behavior in rats, accompanied by increased plasma concentrations of CRP and IL-6. Moreover, the protein expressions of BDNF, Copine 6, and synapsin I were decreased in both the hippocampus and the PFC of CUMS rats, and the protein expression of synaptotagmin I was decreased in the hippocampus. Furthermore, Pearson’s test revealed a potential relationship between the depression-like behavior, the plasma CRP concentration, and the protein expressions of BDNF, Copine 6, synapsin I, or synaptotagmin I in the hippocampus or the PFC. Together with our previous results, the current findings suggest that apart from immune activation, the BDNF-related imbalance of Copine 6 expression in the brain might play a crucial role in stress-associated depression-like behaviors and synaptic plasticity changes.

## Introduction

Depression is one of the most prevalent psychiatric disorders. According to the data provided by the World Health Organization, depression affects approximately 350 million individuals in the world. Currently, most available antidepressant medications are aimed to increase the content of monoamine neurotransmitters in the synaptic cleft, based on the serendipitous discoveries of the clinical efficacy of two classes of antidepressants in the 1950s (Lopez-Munoz and Alamo, 2009). However, it has been reported that these drugs are not effective in all depressed patients and, even if they are, take weeks to months to produce a response (Berton and Nestler, 2006). Although vortioxetine has been reported as a novel antidepressant with multimodal activity and a faster response (Sanchez et al., 2015), targeting the 5-HT3, 5-HT7, 5-HT1, 5-HT1B, and 5-HT1A receptors and the serotonin (5-HT) transporter, this medication requires 8 weeks to reduce the Montgomery–Asberg depression rating scale (MADRS) total score (Mahableshwarkar et al., 2015) and has an adverse event profile similar to that of other selective serotonin reuptake inhibitors (SSRIs) (Zhang et al., 2015). Similarly, the non-competitive, glutamatergic *N*-methyl-D-aspartate receptor antagonist (R,S)-ketamine, exerts rapid and sustained antidepressant effects after a single dose in patients with depression, but its use is also associated with undesirable side effects (Zanos et al., 2016). Therefore, the mechanisms underlying the pathogenesis of depression need to be explored further and new targets for the development of next-generation, rapid-acting antidepressants must be identified (Malinow, 2016; Zanos et al., 2016).

Increasing evidence from animal and human studies show that stressful life events are among the most potent factors that trigger depressive episodes (Swaab et al., 2005), and more attention has been given to the neurobiological mechanisms underlying the association between stress and depression (Muller et al., 2011). The results of our previous studies have demonstrated that chronic unpredictable mild stress (CUMS) induces depression-like behavior and hyperactivity of the hypothalamic–pituitary–adrenal axis in rats, accompanied by imbalances in the leptin signaling pathway and hypothalamic synaptic plasticity (Ge et al., 2013). Moreover, feeding regulation-associated factors (Ge et al., 2015b) and metabolic disease, including subclinical hypothyroidism (Ge et al., 2016) and non-alcoholic fatty liver disease (Chen et al., 2017) also contribute to depression-like behaviors. Thus, the pathogenesis of depression is complicated by multiple risk factors.

Brain-derived neurotrophic factor (BDNF) is a critical effector of depression-like behaviors and antidepressant responses. Involved in neuronal development and neurotransmitter release, synaptic vesicle-associated proteins are indispensable to the integrity of synaptic structure and function. Synaptic vesicle-associated proteins are implicated in the regulation of BDNF-induced axonal growth and neurotransmitter release (Kao et al., 2017; Marte et al., 2017), and increasing evidence has demonstrated the relationship between synaptic dysfunction and depression (Duman and Aghajanian, 2012). Synapsin I is a regulator of synaptic transmission and is believed to affect axonal elongation and branching (Chin et al., 1995), and synaptotagmin I is an integral protein required for vesicle fusion and neurotransmitter release (Greengard et al., 1993). Differential expression of these two proteins may contribute to the molecular basis of stress-induced changes in synaptic plasticity in the hypothalamus (Ge et al., 2013), hippocampus, and cortex (Wu et al., 2007). The interaction between the synaptic vesicle-associated proteins and BDNF might trigger the imbalance of synaptic plasticity that occurs in depression (Kao et al., 2017; Marte et al., 2017).

Copines are a family of cytosolic proteins with the ability to bind to phospholipids in a calcium-dependent manner, which make them interesting candidates for actors in synaptic plasticity. Copine 6 is a neuron-specific member of the copine family. Shortly after Copine 6 was first described, Nakayama et al. reported a correlation between Copine 6 expression and neuronal activity, as indicated by its increased expression either upon long term potentiation (LTP) induction or kainate injection (Nakayama et al., 1998). Subsequently, further studies focused on its role in neuropsychiatric actions. Copine 6 transcripts and protein are expressed in the postnatal brain with peak expression in the hippocampus (Xu et al., 2015), and by regulating hippocampal synaptic plasticity, Copine 6 plays a crucial role in learning and memory (Reinhard et al., 2016). Knockout of Copine 6 induced a deficiency of hippocampal LTP and learning and memory in mice (Xu et al., 2015). More recently, a significantly increased expression of Copine 6 in hippocampal slices was observed after treatment with BDNF (Burk et al., 2018). Considering the crucial role of the hippocampus in the regulation of mood and behaviors, we decided to focus on Copine 6 expression in the hippocampus and the prefrontal cortex (PFC) of CUMS rats.

Dysfunction of the immune/inflammatory response is another contributor to depression (Hodes et al., 2015). Meta-analyses have indicated that C-reactive protein (CRP), interleukin-6 (IL-6), and TNF-α are the most robust evidence-based inflammatory markers associated with depression (Dowlati et al., 2010). Although Chocano-Bedoya et al. (2014) reported no significant correlation between IL-6 or CRP and depression, a growing number of studies have demonstrated that increased plasma IL-6 and CRP levels are positively associated with depression (Liu et al., 2014; Wium-Andersen et al., 2014), and can even predict subsequent depressive symptoms (Valkanova et al., 2013). Consistent with our previous study (Ge et al., 2015b), plasma concentrations of IL-6 and CRP were significantly increased in depressed rats induced by intraperitoneal injection of nesfatin-1.

To gain further insights into the association of stress with depression and to explore the possible change of Copine 6 expression in stressed rats, we replicated the CUMS rat model and observed their depression-like behaviors. Stress-induced alterations of Copine 6 and BDNF expression in the hippocampus and the PFC were detected via western blot. Changes in synaptic plasticity were investigated through the protein expressions of synapsin I and synaptotagmin I after CUMS in the rats’ hippocampus and PFC, and the plasma concentrations of IL-6 and CRP were detected using ELISA commercial kits.

## Materials and Methods

### Animals

Twenty male Sprague-Dawley rats, aged 2 months, were purchased from Anhui Experimental Animal Center of China. The rats were divided randomly into control and CUMS groups and maintained under a 12:12 h light/dark cycle (lights on 07:00 h). The ambient temperature was maintained at 21–22°C with 50–60% relative humidity. Rats in the control group were housed 5 per cage with free access to food and water, while rats in the CUMS group were raised solitarily and received stress according to the CUMS procedure. All experimental procedures in this study were approved by the Animal Care and Use Committee at Anhui Medical University, which complies with the National Institute of Health Guide for the Care and Use of Laboratory Animals (NIH publication No. 85-23, revised 1985).

### CUMS Procedure

The CUMS paradigm consisted of daily exposure to alternating stressors along with occasional overnight stressors for four consecutive weeks. The stressors consisted of (Xu et al., 2015) (1) 24 h of social crowding (10 rats per cage); (2) a 20-min warm swim at 30°C; (3) 24 h in a cage tilted at 30° from the horizontal; (4) a 5-min cold swim at 8–10°C; (5) 24 h in a wet cage; (6) a 2-min tail pinch; and (7) 24 h of food and water deprivation. The different stressors were distributed randomly over an interval of at least 7 days.

### Behavioral Tests

Behavioral tests were performed in a soundproof room with a neutral environment. All the tests were carried out between 08:30 and 12:30 and were matched between the groups. The observers were blind to the treatment. The behavioral tests were monitored and recorded by a digital camera interfaced to a computer running the ANY-maze video imaging software (Stoelting Co., Wood Dale, IL, United States).

**FIGURE 1:**
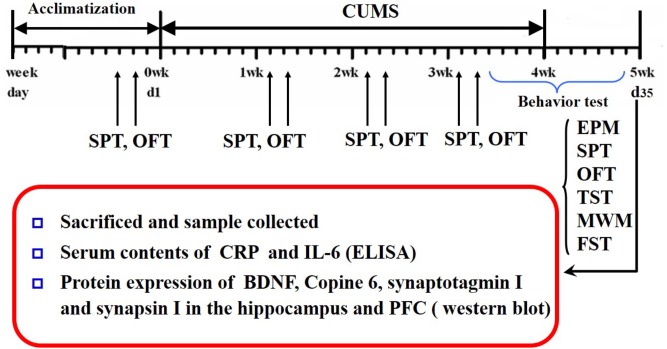
Schedule of the experimental design. CUMS, chronic unpredictable mild stress; OFT, open field test; SPT, saccharin preference test; EPM, elevated plus maze; YM, Y-maze; MWM, Morris water maze; TST, tail suspension test; FST, forced swimming test; CRP, C-reactive protein; BDNF, brain-derived neurotrophic factor; PFC, prefrontal cortex.

Saccharin preference and open-field tests were conducted every week during CUMS. The Morris water maze, elevated plus maze, tail suspension, and forced swimming tests were conducted after CUMS was completed. The schedule is shown in Figure 1.

#### Saccharin Preference Test (SPT)

The SPT is commonly used to assess anhedonic behavior in rodents. After a 12-h period of food and water deprivation, all the rats were given free access to two bottles, one containing plain water and one containing a 1% saccharin solution, After 6 h, the volumes of water and saccharin consumed were measured. The saccharin preference index (SPI), which is the percentage of the saccharin solution that was ingested, was used as a measure of sensitivity to hedonia in rats.

#### Open Field Test (OFT)

The OFT, which provides simultaneous measures of locomotion, exploration, and anxiety, was carried out according to our previous studies (Ge et al., 2014). The apparatus consisted of a black square arena 100 cm × 100 cm with a black wall 30 cm high. The floor was marked with a grid dividing the floor into 16 equal-sized squares. During a 5 min observation period, rats were placed at one corner of the apparatus facing the wall. The distance, duration, and frequency in the center, and the frequencies of rearing, grooming, and defecation were recorded.

#### The Elevated Plus Maze (EPM) Test

The EPM test was designed according to the description in our previous studies (Ge et al., 2014) with little modification. Briefly, the maze (made of Plexiglas) consisted of a plus-shaped apparatus, with two opposite closed arms (45 cm × 11 cm) enclosed with walls (22 cm in height) and two opposite open arms (45 cm × 11 cm, without walls). The whole apparatus had a central arena (11 cm × 11 cm) and was elevated 80 cm above the floor. Each rat was placed in the central arena of the maze facing an open arm and was allowed to explore the maze for 5 min. The distance traveled in the open arms and the closed arms was analyzed.

#### Morris Water Maze (MWM) Test

The MWM test was used to test spatial learning and memory. The pool (1.6 m in diameter) was filled with opaque water and surrounded by complex maze cues. The escape platform (9 cm in diameter) was placed in the center of a designated quadrant with its top positioned 1.3 cm below the water surface. In the place navigation test, each rat received four trials per day of training for 3 days. The rat was allowed 60 s to find the platform, and stayed there for 20 s. If a rat failed to find the hidden platform within 60 s, it was guided to the platform and allowed to remain there for 20 s. A probe test was conducted on day 4, in which the hidden platform was removed and the rat was allowed to swim for 60 s. The escape latency to find the hidden platform in the place navigation test and the duration of time spent by the rats in the target quadrant in the probe trial were analyzed.

#### Tail Suspension Test (TST)

The TST was carried out according to the method described in our previous studies (Ge et al., 2013). Briefly, rats were suspended by bands around their tails and hung from a mounted hook 50 cm above the floor for 6 min. Time spent immobile during the last 4 min was measured. Immobility time was defined as a lack of all movement except for whisker movement and respiration.

#### Forced Swimming Test (FST)

The FST was carried out according to the method described in our previous study (Ge et al., 2013). The behavioral cylinder was 60 cm high and 25 cm in diameter, maintained at 24–25°C, and filled with 30 cm of water. The FST paradigm includes two steps: an initial 15 min pretest followed by a 5 min test 24 h later. The rats were considered to be immobile when they did not make any active movements. Struggling was considered to occur when the rats made active movements with their forepaws in and out of the water along the side of the swim chamber. Swimming was considered to occur when the rats made active swimming or circular movements.

### Western Blot Assays

The hippocampus and the PFC from four rats in each group were rapidly dissected, frozen in liquid nitrogen, and stored at -80°C. The tissues were homogenized in radioimmunoprecipitation assay (RIPA) buffer (50 mM Tris-HCl, pH 7.4, 0.1% SDS, 1% NP-40, 0.25% sodium deoxycholate, 150 mM NaCl, 1 mM EDTA, 1 mM EGTA, and 1 mM Na_3_VO_4_). Before homogenization, a protease inhibitor cocktail (Roche, Indianapolis, IN, United States) and the phosphatase inhibitor PhosSTOP (Roche, Indianapolis, IN, United States) were added. Protein quantitation was conducted using a Lowry Protein Assay Kit (Meiji Biotech. Co., LTD., Shanghai, China). The same quantity (∼50 μg) of protein from each animal was loaded and separated by 15% SDS-PAGE and then transferred onto a polyvinylidene difluoride membrane (Amersham Biosciences, United Kingdom). The membrane was blocked with 5% skim milk for 1 h; incubated with antibodies targeting BDNF (1:1000; ImmunoWay, Newark, DE, United States), Copine 6 (1:1,000; Santa Cruz Biotechnology, Inc., Santa Cruz, CA, United States), synapsin I (1:1000; ImmunoWay, Newark, DE, United States), synaptotagmin I (1:1000; ImmunoWay, Newark, DE, United States), or β-actin (1:1000; Zhongshan Biotechnology, INC, Beijing, China) at 4°C overnight; and then incubated with a horseradish peroxidase-conjugated secondary antibody (1:2000) at 37°C for 2 h. The blots were developed with the Easy Enhanced Chemiluminescence Western Blot Kit (Pierce Biotechnology, Rockford, IL, United States). Protein bands were scanned and analyzed using Image J software (NIH), and the protein expression was normalized to β-actin.

### Measurement of the Plasma Concentrations of IL-6 and CRP

Twenty-four hours after the last behavioral test, the rats were deeply anesthetized with chloral hydrate, and blood was taken from the abdominal aorta. The plasma was collected, and the concentrations of IL-6 and CRP were measured using commercially available enzyme-linked immunosorbent assay (ELISA) kits (Yuanye Biotech. Co., LTD., Shanghai, China) according to the manufacturer’s instructions.

### Statistical Analyses

All statistical analyses were performed using SPSS (version 12.0.1, SPSS Inc., Chicago, IL, United States). Data are expressed as the means ± SEM and *P* < 0.05 was considered statistically significant. The effect of time and stress on the bodyweight-gain and the behavior of the rats in the OFT and the SPT were analyzed using repeated measures ANOVA. The effect of training and stress on escape latency in the MWM test was also analyzed using repeated measures ANOVA. The difference in other parameters between the control and CUMS groups was tested by using Student’s *t-* test. The correlation analysis was performed by Pearson’s correlation test.

## Results

### Slow Increase of Body-Weight Induced by CUMS

**FIGURE 2:**
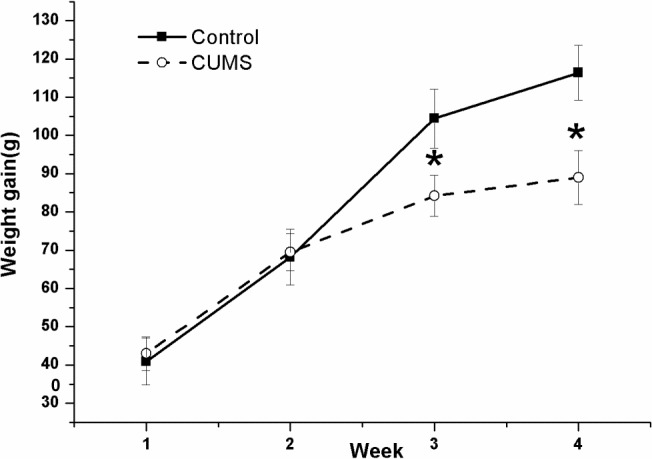
Body-weight gain in the control and CUMS rats. The data are presented as the mean ± SEM, with 10 rats in each group. The net body-weight gain was lower in the CUMS group than in the control group during the latter 2-week stress period. ^∗^*P* < 0.05, ^∗∗^*P* < 0.001; compared with control group.

Figure 2 shows the changes in body-weight gain during the four consecutive weeks in the two groups. The result of repeated measures ANOVA showed that time [(*F*(3,54) = 147.068, *P* < 0.001)] but not stress [(*F*(1,18) = 1.83, *P* = 0.189)] had a significant effect on body-weight gain, with an interactive effect between time and stress [(*F*(3,54) = 11.031, *P* < 0.001)]. The net bodyweight-gain was lower in the CUMS group than in the control group during the latter 2-week stress period.

### Decrease of Locomotor Activity and Exploration Behavior Induced by CUMS

**FIGURE 3:**
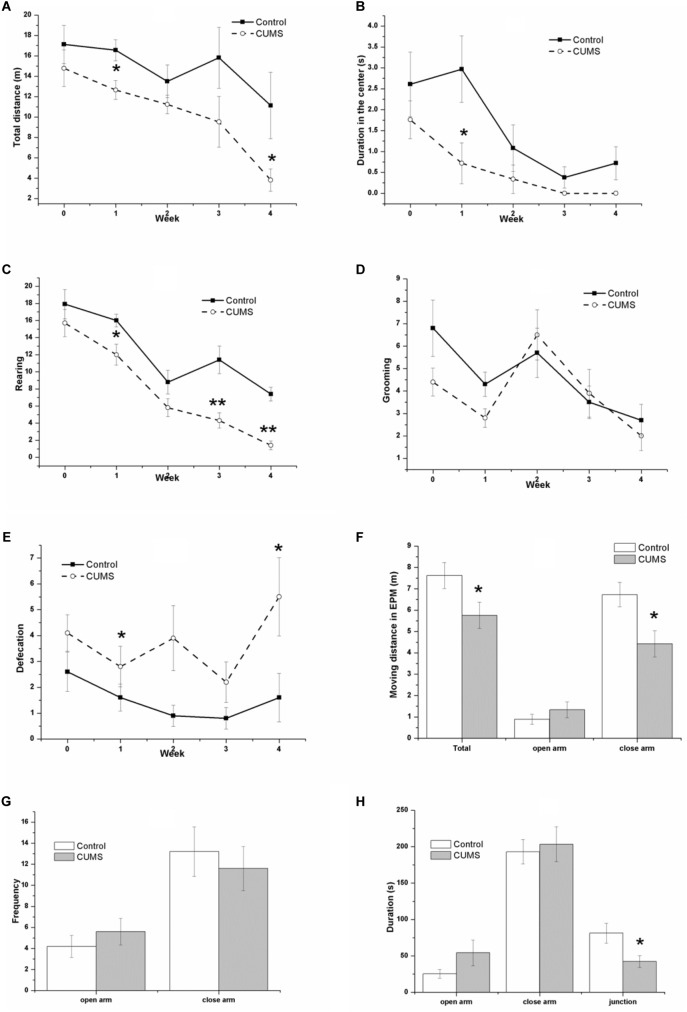
Behavior of the control and CUMS rats in the OFT and the EPM tests. The data are presented as the mean ± SEM, with 10 rats in each group. In the OFT **(A–E)**, repeated measures ANOVA show that both time and stress had a significant effect on total moving distance **(A)**, center duration **(B)**, and rearing number **(C)**. The time effect on the grooming number **(D)** and the stress effect on the defecation number **(E)** are also shown. In the EPM test **(F–H)**, the total distance **(F)**, distance in the closed arm **(F)**, and duration in the junction **(H)** in the CUMS group were less than those in the control group, although there is no significant difference in the distance **(F)**, frequency **(G)**, and duration **(H)** in the open arm between groups, ^∗^*P* < 0.05, ^∗∗^*P* < 0.001; compared with control group.

Figure 3 shows the performance of rats in the OFT and the EPM test. In the OFT, the result of repeated measures ANOVA showed that both time [*F*(4,72) = 6.358, *P* < 0.001] and stress [*F*(1,18) = 7.753, *P* = 0.012] had significant effects on the total moving distance (Figure 3A), without an interactive effect between time and stress [*F*(4,72) = 0.806, *P* = 0.525]. Consistently, both time [*F*(4,72) = 7.488, *P* < 0.001] and stress [*F*(1,18) = 8.389, *P* = 0.010] had significant effects on the center duration (Figure 3B), without an interactive effect between time and stress [*F*(4,72) = 1.218, *P* = 0.311]. Both time [*F*(4,72) = 36.089, *P* < 0.001] and stress [*F*(1,18) = 32.058, *P* < 0.001] had significant effects on the rearing number (Figure 3C), without an interactive effect between time and stress [*F*(4,72) = 1.436, *P* = 0.231]. However, an effect of time [*F*(4,72) = 7.267, *P* < 0.001] but not stress [*F*(1,18) = 1.745, *P* = 0.149] was identified for the number of grooming movements (Figure 3D), without an interactive effect between time and stress [*F*(4,72) = 0.015, *P* = 0.903]. In contrast, it was demonstrated that stress [*F*(1,18) = 9.749, *P* = 0.006] but not time [*F*(4,72) = 2.222, *P* = 0.075] had a significant effect on the defecation number (Figure 3E), without an interactive effect between time and stress [*F*(4,72) = 1.094, *P* = 0.366].

In the EPM test, the CUMS rats traveled a smaller distance than the control rats did, with significant differences between the groups in the total distance (Figure 3F), the distance in the closed arm (Figure 3F), and the duration in the junction (Figure 3H). There were no significant changes in the distance in the open arm (Figure 3F) or in the frequency (Figure 3G) or duration (Figure 3H) in both the open and closed arm between groups.

### Anhedonia, Despair-Behavior, Impaired Learning, and Memory Ability Induced by CUMS

**FIGURE 4:**
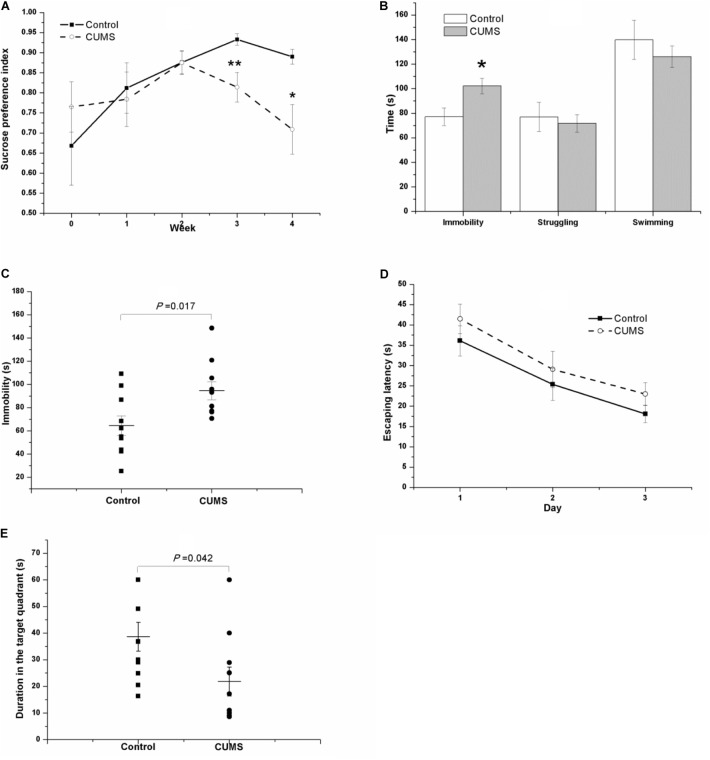
Behavior of the control and CUMS rats in the SPT, TST, FST, and MWM test. The data are presented as the mean ± SEM, with 10 rats in each group. In the SPT **(A)**, the results of repeated measures ANOVA show an effect of time but not stress on the saccharin preference index from week 0 to stress exposure week 2. From the second to the fourth stress exposure weeks, both time and stress had a significant effect on the saccharin preference index. Compared with the control rats, the CUMS rats spent more time immobile in the FST **(B)** and TST **(C)** but less time in the probe phase of the MWM test **(E)**. In the place navigation of the MWM test, the escape latency of both groups in the consecutive three days’ place navigation test gradually declined **(D)**, with no significant difference between groups.

As shown in Figure 4A, the tendency of the saccharin preference index in the CUMS group is different from that in the control group. Because they are almost identical in the second week (0.876 ± 0.028 of control rats vs. 0.875 ± 0.029 of CUMS rats), the data were analyzed separately. From week 0 to week 2 of the CUMS, results of repeated measures ANOVA showed that time [*F*(2,36) = 3.718, *P* = 0.034] but not stress [*F*(1,18) = 0.152, *P* = 0.701] had a significant effect on the saccharin preference index, without an interactive effect between time and stress [*F*(2,36) = 0.636, *P* = 0.535]. However, as for the duration from the second week to the fourth week of stress exposure, both time [*F*(2,36) = 3.973, *P* = 0.028] and stress [*F*(1,18) = 8.460, *P* = 0.009] affected the saccharin preference index, with an interactive effect between time and stress [*F*(2,36) = 4.427, *P* = 0.019].

In the FST (Figure 4B) and the TST (Figure 4C) after 4 weeks of CUMS, the CUMS rats spent a longer time immobile.

In the MWM task, the escape latency of both groups in the consecutive 3-day place navigation test declined gradually (Figure 4D). Although the CUMS rats seemed to spend slightly more time in finding the hidden platform than the control rats did, the results of repeated measures ANOVA showed that training [*F*(2,36) = 29.181, *P* < 0.001] but not the stress [*F*(1,18) = 1.273, *P* = 0.274] had a significant effect on escape latency, and no interactive effect was found between time and stress [*F*(2,36) = 0.068, *P* = 0.934]. However, in the probe phase of the MWM test, the duration in the target quadrant of the CUMS rats was shorter than that in the control rats (Figure 4E).

### Imbalanced Expression of BDNF, Copine 6, Synapsin I, and Synaptotagmin I in the Hippocampus and the PFC Induced by CUMS

**FIGURE 5:**
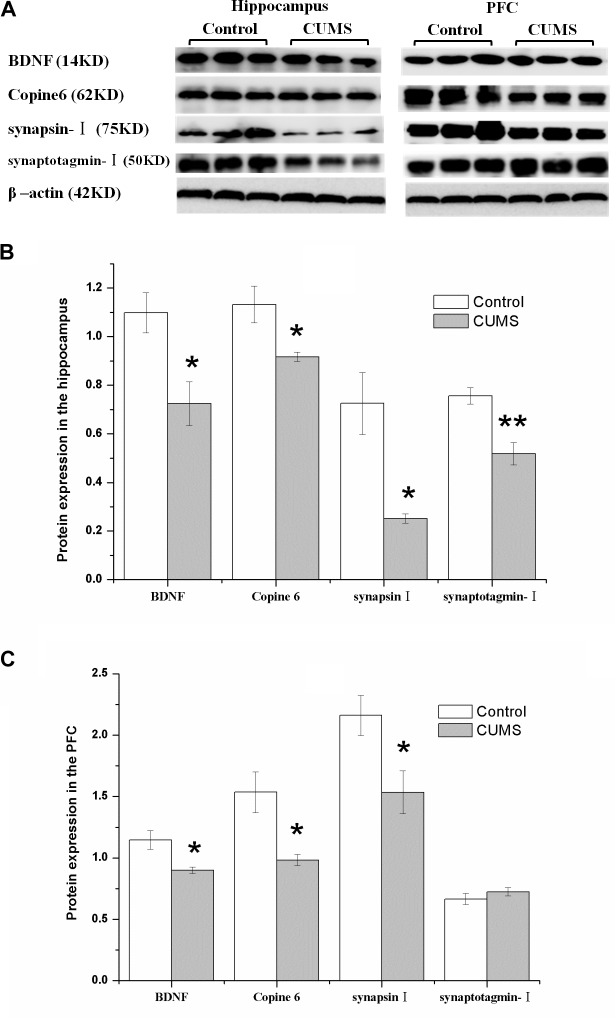
Protein expressions of BDNF, Copine 6, synapsin I, and synaptotagmin I in the hippocampus and the PFC of the control and CUMS rats. Typical graph **(A)** and statistical analyses **(B,C)** of the western blot results. Data in **(B,C)** are presented as the mean ± SEM (*n* = 4 for each group). In the hippocampus **(A,B)**, the expression of all four proteins was all decreased in the CUMS group. In the PFC **(A,C)**, remarkable decreases in the BDNF, Copine 6 and synapsin I protein expressions were observed, with an insignificant change in synaptotagmin I expression. ^∗^*P* < 0.05, ^∗∗^*P* < 0.001; compared with control group.

**FIGURE 6:**
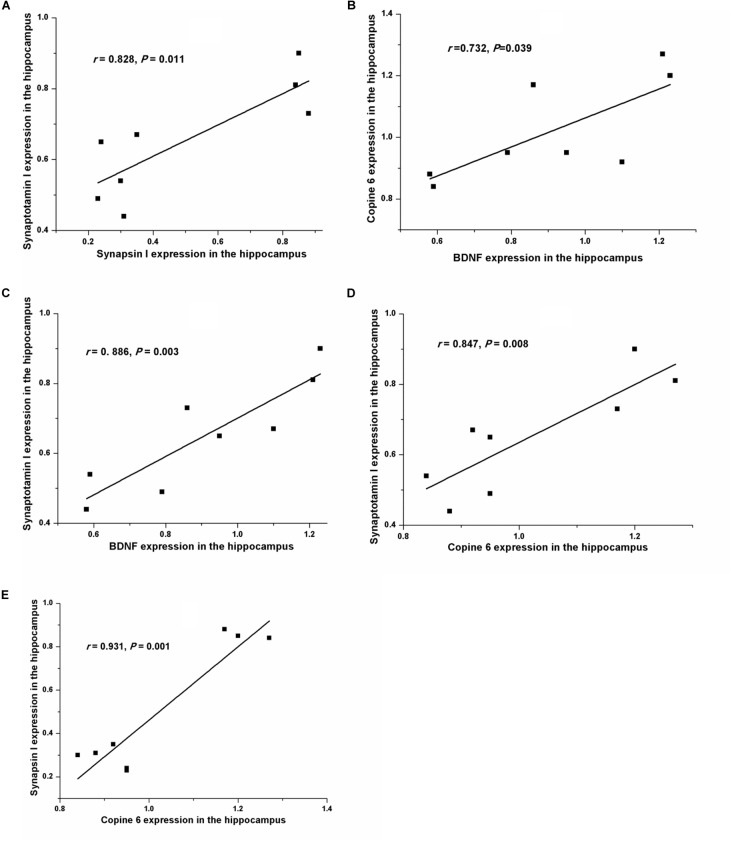
Correlation analysis of the protein expressions of BDNF, Copine 6, synapsin I, and synaptotagmin I in the hippocampus and the PFC. In the hippocampus, apart from the positive correlation between synapsin I and synaptotagmin I **(A)** expression, BDNF expression was positively related to the expression of Copine 6 **(B)** and synaptotagmin I **(C)**. Additionally, the protein expression of Copine 6 was positively correlated with that of synaptotagmin I **(D)** and synapsin I **(E)**.

Figure 5 shows the protein expressions of BDNF, Copine 6, synapsin I, and synaptotagmin I in the hippocampus and the PFC of the rats in both groups. In the hippocampus, the expression of all four of these four proteins was decreased in the CUMS group. Apart from the predicable positive correlation between synapsin I and synaptotagmin I (*r* = 0.828, *P* = 0.011, Figure 6A), a positive relationship was also found between the expression of BDNF and Copine 6 (*r* = 0.732, *P* = 0.039, Figure 6B) and synaptotagmin I (*r* = 0.886, *P* = 0.003, Figure 6C). Additionally, the hippocampal protein expression of Copine 6 was positively correlated with that of synaptotagmin I (*r* = 0.847, *P* = 0.008, Figure 6D) and synapsin I (*r* = 0.931, *P* = 0.001, Figure 6E).

Consistently, there was a remarkable decrease in the BDNF, Copine 6, and synapsin I protein expressions in the PFC of CUMS rats, although the expression of synaptotagmin I was not significantly changed.

**FIGURE 7:**
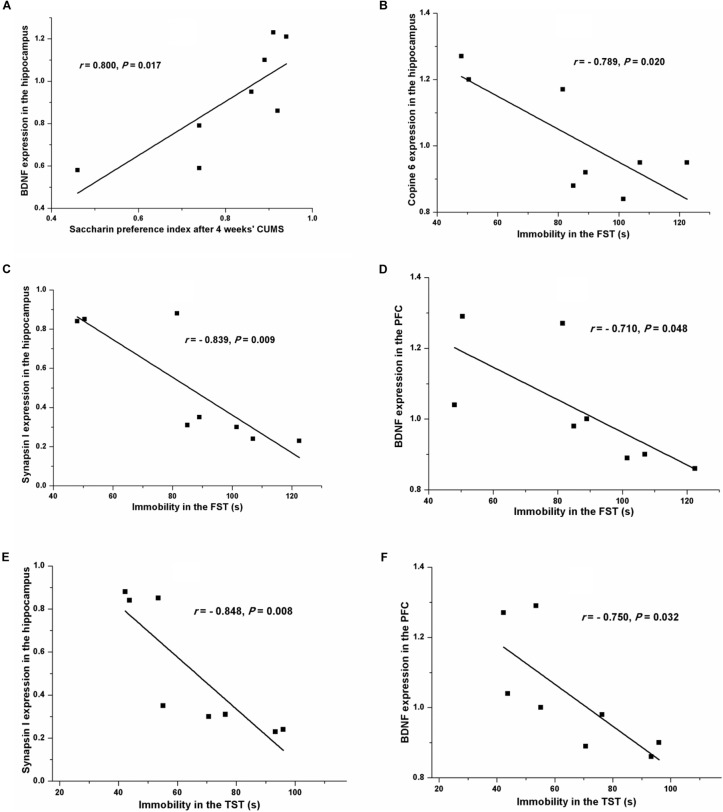
Correlation analysis of the depression-like behavior and the protein expressions of BDNF, Copine 6, or synapsin I in the hippocampus and the PFC. A positive relationship was found between the saccharin preference index after four consecutive weeks of CUMS and the hippocampal expression of BDNF **(A)**. A negative relationship was seen between immobility in the FST and the expression of Copine 6 **(B)** and synapsin I **(C)** in the hippocampus and the expression of BDNF in the PFC **(D)**. Immobility in the TST was negatively related to the hippocampal expression of synapsin I **(E)** and the expression of BDNF in the PFC **(F)**.

Pearson’s test revealed a positive relationship between the saccharin preference index after four consecutive weeks of CUMS and the hippocampal expression of BDNF (*r* = 0.800, *P* = 0.017, Figure 7A). A negative relationship was seen between immobility in the FST and the expression of Copine 6 (*r* = -0.789, *P* = 0.020, Figure 7B) and synapsin I (*r* = -0.839, *P* = 0.009, Figure 7C) in the hippocampus and the expression of BDNF in the PFC (*r* = -0.710, *P* = 0.048, Figure 7D). Likewise, immobility in the TST was also found to be negatively related to the hippocampal expression of synapsin I (*r* = -0.848, *P* = 0.008, Figure 7E) and the expression of BDNF in the PFC (*r* = -0.750, *P* = 0.032, Figure 7F). However, immobility in the TST was not remarkably related to the expression of Copine 6 protein in the hippocampus (*r* = -0.682, *P* = 0.063).

### Increase of Plasma IL-6 and CRP Concentrations Induced by CUMS

**FIGURE 8:**
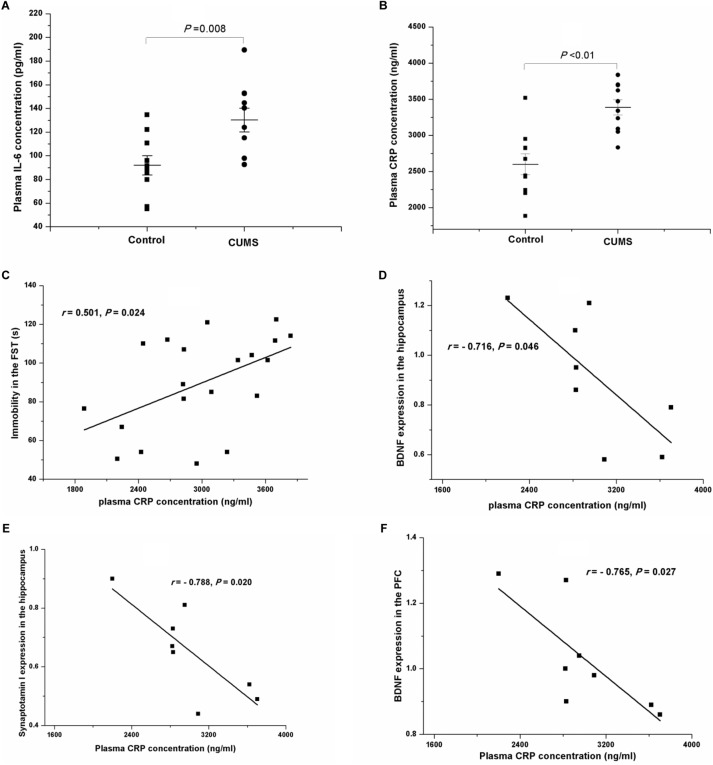
Plasma IL-6 and CRP concentrations of the control and CUMS rats, and correlation analysis of the plasma CRP concentration and immobility in the FST and the protein expressions in the hippocampus and the PFC. The data are presented as the mean ± SEM in **(A,B)**, with 10 rats in each group. The plasma IL-6 **(A)** and CRP **(B)** concentrations were both notably increased in the CUMS group. The plasma CRP concentration was positively related to immobility in the FST **(C)**, and negatively related to the protein expressions of BDNF **(D)** and synaptotagmin I **(E)** in the hippocampus and BDNF in the PFC **(F)**.

After a 4-week period of CUMS, the plasma concentrations of IL-6 (Figure 8A) and CRP (Figure 8B) were both remarkably increased, compared with those of the control rats. Interestingly, the results of Pearson’s test showed that the plasma concentrations of CRP were positively related to the immobility in the FST (*r* = 0.501, *P* = 0.024, Figure 8C) but negatively correlated to the protein expressions of BDNF (*r* = -0.716, *P* = 0.046, Figure 8D) and synaptotagmin I (*r* = -0.788, *P* = 0.020, Figure 8E) in the hippocampus, or to the protein expression of BDNF in the PFC (*r* = -0.765, *P* = 0.027, Figure 8F).

## Discussion

In the present study, we duplicated a CUMS-induced depression rat model, observed the behavior of the animals, and explored the possible mechanisms underlying the changed behavioral performance. Our results verified the depression-like behaviors induced by CUMS and demonstrated that the protein expressions of BDNF, Copine 6, and synapsin I were significantly decreased in both the hippocampus and the PFC of CUMS rats, together with decreased hippocampal expression of synaptotagmin I. Moreover, the plasma levels of IL-6 and CRP were remarkably increased after 4 weeks of CUMS. Furthermore, our results demonstrated the potential relationships among depression-like behavior, imbalanced protein expression in the related brain areas, and increased immune activity. These alterations are in accordance with the network hypothesis of depressive disorders that proposes that the compromised functionality of relevant neural networks may underlie the development of depressive symptomatology (Djordjevic et al., 2012).

Consistent with our previous findings (Ge et al., 2013), the present study shows that chronic unpredictable stress induces depression-like behavioral deficits in rats, including anhedonia, reduced locomotor activity and exploration behavior, and “behavioral despair,” as indicated by the decreased saccharin preference index, decreased locomotion and rearing in the OFT, and increased immobility in both the FST and the TST. In accordance with the finding that an anxiety-/depression-like phenotype is associated with a cognitive deficit (Darcet et al., 2014), CUMS rats showed an impairment in learning and memory ability, as indicated by the shorter duration in the target quadrant in the probe test of the MWM test, although the increased escape latency was only a tendency without a significant difference between groups.

Although little is known about the molecular components and mechanisms involved in the stress response, increasing evidence suggests that BDNF-associated synaptic dysfunction is a key pathophysiological hallmark in depression. Prolonged stress has been associated with region-specific changes in the expression of BDNF, synaptotagmin I, and synapsin I (Smith et al., 1995; Thome et al., 2001), and decreased BDNF has been associated with age-related hippocampal dysfunction, memory impairment, and increased risk for depression (Erickson et al., 2012; Platenik et al., 2014). In consistence, our results showed that the protein expressions of BDNF and synapsin I were decreased in the hippocampus and the PFC of CUMS rats, along with a decline in the synaptotagmin I expression in the hippocampus. BDNF plays a crucial role in the regulation of synaptic function in a site-specific manner (Leal et al., 2014; Chang et al., 2018), and synapsin I plays a pivotal role in BDNF signal transduction during axonal growth (Marte et al., 2017). In our previous study (Ge et al., 2015a), decreased protein expressions of BDNF and synapsin I were also found in a subclinical hypothyroidism model with a positive relationship. These findings suggest a remarkable linkage between BDNF and synaptic structure and function. In our present study, Pearson’s test uncovered a positive relationship between the hippocampal expression of synaptotagmin I and BDNF, providing new evidence for the close link between BDNF and the synaptotagmin family in regulating synaptic plasticity. Although the exact mechanism remains unknown, BDNF may serve as a homeostatic regulator, eliciting neuroprotective functions when neurons are damaged in disease conditions (Lu et al., 2013). Synapsins could act by regulating the ratio of lipids in intracellular membranes, thereby promoting lipid raft formation, and regulating BDNF-mediated synaptic potentiation and axon elongation (Kao et al., 2017). Thus, a rational way to conduct a future experiment would include a combination of activating the BDNF pathway and using a more reliable and sensitive method to measure the resulting synaptic changes.

Copine 6 is a brain-specific, calcium-dependent, phospholipid-binding protein with a scarcely described function (Cowland et al., 2003; Walf and Frye, 2007). Based on its ability to bind, activate, and recruit the Rho GTPase Rac1 to cell membranes, Copine 6 plays a vital role in the regulation of hippocampal synaptic plasticity (Reinhard et al., 2016); consistent with this idea, Copine 6-knockout mice present impairments in learning and memory abilities. The decreased expression of Copine 6 in the hippocampus and the PFC of rats with non-alcoholic fatty liver disease was demonstrated in our previous study (Chen et al., 2017). In consistence, the present results showed a decreased expression of Copine 6 in the hippocampus and the PFC of CUMS rats. Moreover, in accordance with the report that BDNF treatment increases the expression of Copine 6 in hippocampal slices (Burk et al., 2018), a positive relationship was found between the hippocampal expression of BDNF and Copine 6 in the present study. Additionally, the protein expression of Copine 6 in the hippocampus was also positively correlated with that of synaptotagmin I and synapsin I. These findings indicate again the important role of Copine 6 in the regulation of hippocampal synaptic plasticity. Although the mechanism remains to be explored, it is rational to propose a role of BDNF in connecting the changes in hippocampal Copine 6 expression and synaptic plasticity.

Additionally, Pearson’s test showed the potential connection between depression-like behavior and the changes in the protein expression in the hippocampus and the PFC. The saccharin preference index, which is used as an indicator of anhedonia, was positively related to the hippocampal BDNF expression. Moreover, despair behavior, as indicated by immobility in the FST or the TST, was negatively related to the expression of synapsin I in the hippocampus and the expression of BDNF in the PFC. However, the hippocampal expression of Copine 6 was negatively related to immobility in the FST but not in the TST. These results might be ascribed to the hypothesis that different behaviors are controlled by different brain areas.

A growing body of evidence suggests the potential relationships between immune hyperactivity and the severity of the symptoms of depression (Liu et al., 2014; Wium-Andersen et al., 2014). Increased plasma concentrations of IL-6 and CRP have been detected in depressed patients and animals (Voorhees et al., 2013; Liu et al., 2014; Wium-Andersen et al., 2014). In accordance with these findings, our results show increased plasma IL-6 and CRP levels in the CUMS rats, and a positive relationship between plasma CRP concentration and immobility in the FST. Moreover, Pearson’s test showed that plasma CRP concentrations were negatively related to the protein expression of BDNF in the hippocampus and the PFC. These findings further support the crucial role of immune activity in the pathogenesis of stress-induced depression.

Taken together, our study replicated depression-like behavior in a rat CUMS model, and decreased the expression of BDNF and its related synaptic plasticity change in the hippocampus and the PFC, accompanied by hyperactivity of the immune response. Moreover, our study showed a BDNF-related decrease of Copine 6 protein expression in the hippocampus and the PFC of CUMS rats, providing a preliminary evidence for the important role of Copine 6 in the regulation of neuropsychiatric behaviors and synaptic plasticity. These findings might shed light on the pathogenesis of stress-associated depression.

## Author Contributions

J-fG designed the study, and wrote the protocol and the first draft of the manuscript. Y-xH and CT managed the literature searches and the statistical analyses. Y-xH, X-rG, L-lW, and F-hJ performed animal model experiments. KF, X-xC, CW, and ZC performed the gene expression experiments and wrote parts of the manuscript. All authors contributed to and have approved the final manuscript.

## Conflict of Interest Statement

The authors declare that the research was conducted in the absence of any commercial or financial relationships that could be construed as a potential conflict of interest.
